# Study of coordinated development of county urbanization in arid areas of China: The case of Xinjiang

**DOI:** 10.1371/journal.pone.0276235

**Published:** 2022-10-14

**Authors:** Maliyamuguli Abulimiti, Zibibula Simayi, Shengtian Yang, Ziyuan Chai, Yibo Yan

**Affiliations:** 1 College of Geography and Renmote Sensing Science, Key Laboratory of Smart City and Environmental Modeling, Key Laboratory of Oasis Ecology Ministry of Education, Xinjiang University, Urumqi, Xinjiang, China; 2 School of Geography and Remote sensing Science, Beijing Normal University, Beijing, China; Curtin University, AUSTRALIA

## Abstract

Urbanization is a comprehensive process of mutual influence among the population, economy, society and living environment, and it depends on the synergy of a series of factors. This paper uses the statistical data of 76 counties in Xinjiang from 1996 to 2018 to construct a comprehensive urbanization evaluation system. Based on the entropy method, comprehensive evaluation model and coupling coordination model, from the scales of time and space, this paper discusses the current situation of the coordinated development of population, economy, society and living environment factors in counties in Xinjiang in the process of urbanization. Local spatial autocorrelation analysis is used to further study the spatial agglomeration effect of the coupling and coordination of urbanization development in the counties. The results show the following: (1) The comprehensive urbanization level of 76 counties in Xinjiang has the characteristics of "center-periphery" development, and high-level counties are clustered on the northern slopes of the Tian Mountains. (2) Most counties are in a serious state of imbalance; notably, the degree of population-economy-society-living environment coupling and coordination in the border counties and towns is in an unsatisfactory state. (3) The county-level cities in Northern Xinjiang belong to the diffusion and spillover areas, the county-level cities in southern Xinjiang belong to the polarization benefit areas, and most other counties are in the state of no spillover effect.

## Introduction

Urbanization refers to the systematic process of population agglomeration and the continuous expansion of land use scale [1]. It is also a dynamic [2], multifactor, complex social spatial process [3–4]. With rapid economic growth, China’s urbanization has been able to develop rapidly [5], and the degree of urbanization is high in the north and low in the south [6]. This remarkable feature is mainly due to regional economic differences [7]. At the urbanization work conference held by the Central Committee and the National Congress pointed out that urbanization is a strong support for promoting coordinated regional development. Along with the steady development of the level of population urbanization, the level of economic, social and living environment construction should be improved. The main contradiction in Chinese society has been transformed into the contradiction between the people’s growing demand for a better life and unbalanced and inadequate development. Rapid economic development has brought about problems such as low development quality and lack of coordination in some remote areas [8]. In the first year of the 14th Five-Year Plan for Economic and Social Development of the People’s Republic of China (the 14th Five-Year Plan), China’s "Key Tasks for New Urbanization and Urban-Rural Integration Development in 2021" proposed promoting urbanization with the county as an important carrier to accelerate the promotion of urban-rural integration and balanced development. Therefore, county development is also on the agenda of China’s comprehensive promotion of the coordinated development of urbanization. Obviously, the urbanization development of comprehensive population, economy, society and environment is the trend of future development, and at this stage, considerable attention should be paid to the development and potential of county and rural areas [9]. In terms of the development process of the world’s counties, as early as the 1930s, Europe carried out the development of counties based on agricultural tourism; Germany redrawn 237 counties in the 1970s; Japan has become the first country in East Asia to realize the modernization of counties; South Korea, the Netherlands, Israel and other countries have also realized the modernization of the county [10]. Therefore, it can be said that the experiences and lessons of local government reform and county development at home and abroad are of great relevance and have a certain reference value for each other.

Xinjiang, located in the western region of China, is advancing with the opening-up of China, the great development of the western region and the continuous advancement of the “Belt and Road” construction [11, 12], Xinjiang has changed from a relatively closed inland region to the forefront of opening to the outside world, it has become the bridgehead of China’s "Silk Road Economic Belt" and an effective regional cooperation platform between China and relevant counties. By the end of 2018, Xinjiang had established 4 prefecture-level cities and 87 counties (including cities of Xinjiang Production and Construction Corps), with a total year-end population of 18.64 million, accounting for 81.63% of the total population of Xinjiang. The GDP of the counties was 64.47 million, accounting for 52.84% of the GDP of Xinjiang. Due to the influence of the unique natural environment and human factors, there are also great regional differences in the level and degree of urbanization in Xinjiang. As the basic economic unit of China’s current urbanization construction [13], the coordinated development of Xinjiang’s counties is of great significance to urbanization across the whole of Xinjiang Province and even the development of western China.

In the early stage of urbanization research from 1970 to the early 20th century, the degree of urbanization development was measured by indicators such as the proportion of urban population [14], nonagricultural population, and urban construction land [15]. With the imbalance and hierarchy of the relationship between people and land in the traditional extensive urbanization process, the gap in urban population distribution has increased, and the rapid expansion of urban construction has caused damage to the ecological environment. However, the current era of the new economy and the process of sustainable development have caused fundamental changes in the dynamic mechanism of urban development [16], a variety of factors promoting the development of cities and towns, and different dominant forces at different stages of development [17]. Therefore, based on population as a single indicator, the economic, social, environmental and other multidimensional systems in the process of urbanization, as well as their coordinated development status, can reflect the rich connotations of modern urbanization more objectively, comprehensively and scientifically [18]. In recent years, scholars have proposed a variety of comprehensive indicators to describe the level and scale of the coordinated development of urbanization, and the research content has focused on the interactive relationships between urbanization and the ecological environment [19, 20], urbanization and the economy [21, 22], urbanization and industry [23, 24], and urbanization and tourism [25, 26]. However, the index system related to comprehensive urban development established in these studies tends to express the coordination effect between urbanization and other systems and cannot accurately express the coordinated development process within urbanization. Moreover, most of the research on the development of urbanization in Xinjiang takes the prefecture level [27, 28] or city level [29, 30] as the research area, and the research content focuses on the county-level economy [31, 32] and industrial structure [33, 34]. It is obvious that the existing studies tend to study the coordinated development of urbanization in Xinjiang from a macro perspective. Few studies have been conducted on the coupled and coordinated spatiotemporal development of population, economy, society and living environment in the counties in the arid areas of Western China. Moreover, a relatively unified view has not been formed on the selected indicator system. Therefore, based on the former research, this article uses 24 composite indicators, such as total population, urban population, GDP, the proportion of industry in total output value, average wages of on-the-job employees, domestic waste removal, green space rate in built-up areas, and public green space per capita in the process of urbanization in the county, On the basis of making full use of the existing statistical data, the coupling and coordinated development of the four systems of urbanization in various counties in Xinjiang from 1996 to 2018 was explored in both time and space, and whether the development levels of population, economy, society and living environment in various counties in Xinjiang were coordinated was discussed. The quantitative analysis and research results of this paper will enrich the research contents of the coupled and coordinated development of various systems in the process of urbanization development in arid areas of China to a certain extent, and it also has certain reference value for countries with the same regional environment and urbanization development model.

## Study area and methods

### Study area

**Fig 1:**
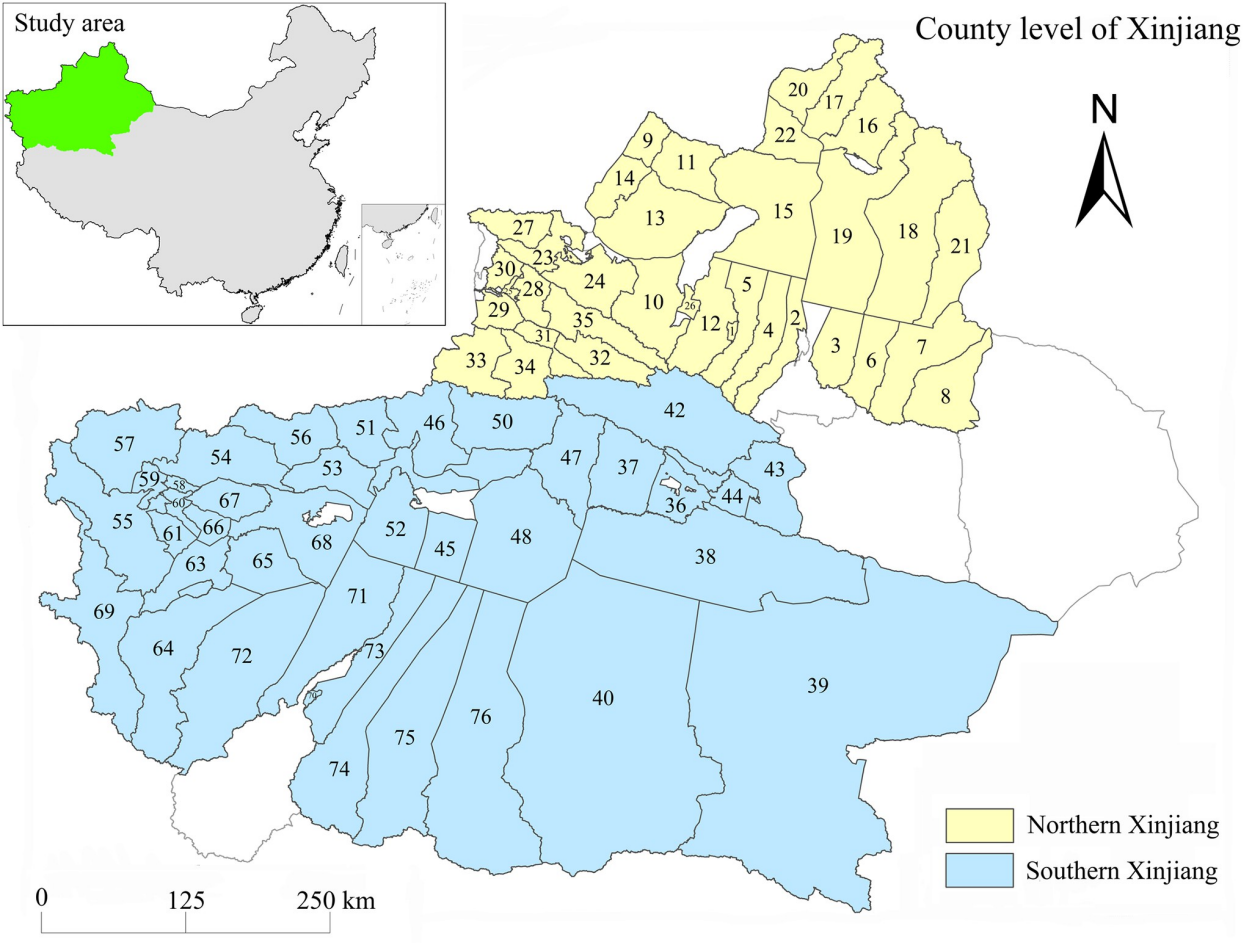
Study area. ^a^ The labels in the Xinjiang county (XJC) map indicate the following (county level cities are indicated by *, and the rest are counties): 1. Shihezi*, 2. Changji*, 3. Fukang*, 4. Hutubi, 5. Manas, 6. Jimsar, 7. Qitai, 8. Mori, 9. Tacheng City, 10. Usu City, 11. Emin, 12. Shawan, 13. Toli, 14. Yumin, 15. Hoboksar, 16. Altay*, 17. Burqin, 18. Fuyun, 19. Fuhai, 20. Habahe, 21. Qinghe, 22. Jeminay, 23. Bole*; 24. Jinghe, 25. Yining*, 26. Kuytun, 27. Wenquan, 28. Yining, 29. Qapqal, 30. Huocheng, 31. Gongliu, 32. Xinyuan, 33. Zhaosu, 34. Tekes, 35. Nilka, 36. Korla*, 37. Luntai, 38. Yuli, 39. Ruoqiang, 40. Qiemo, 41.Yanqi, 42. Hejing, 43. Hoxud, 44. Bohu, 45. Aksu*, 46. Wensu, 47. Kuqa, 48. Xayar, 49. Xinhe, 50. Baicheng, 51. Wushi, 52. Awati, 53. Keping, 54. Artux*, 55. Akto, 56. Akqi, 57. Wuqia, 58. Kashkar*, 59. Shufu, 60. Yingisar, 61. Shule, 62. Zepu, 63. Shache, 64. Yecheng, 65. Makit, 66. Yopurga, 67. Jiashi, 68. Bachu, 69. Tashkorgan, 70. Hotan*, 71. Moyu, 72. Pishan, 73. Lop, 74. Qira, 75. Yutian, 76. Minfeng.

Located in the hinterland of the Eurasian continent, Xinjiang Province is the province with the most border countries in China, the core channel of the Silk Road Economic Belt [35], and a typical representative of the inland region in northwest China [36]. Limited by the special climate and low precipitation natural environment, Xinjiang has a vast territory, a sparse population, a weak economy, and the urban layout is mainly composed of most counties [37, 38]. This paper focuses on 76 counties in Xinjiang (including county-level cities and counties) and divides them into northern and southern Xinjiang regions for analysis, as shown in Fig 1. The Northern Xinjiang Region includes Shihezi City, Turpan City, Hami City, Changji Hui Autonomous Prefecture, Yili Region, Tacheng Region, Altay Region and Bortala Mongolian Autonomous Prefecture. Southern Xinjiang includes Bayingoleng Mongolian Autonomous Prefecture, Aksu Region, Kizilsu Kirgiz Autonomous Prefecture, Kashgar Region and Hotan Region. Considering that Urumqi, Karamay, Turpan, and Hami are prefecture-level cities, and statistical data are not available for Hotan County, Horgos City, Alashankou City, Alar City, Tumshuk City, Wujiaqu City, Beitun City, Tiemenguan City, Shuanghe City, Kekedala City and Kunyu City, these areas are not included in the study.

### Methods

#### Data sources

Taking into account the basic, comprehensive, scientific and dynamic basis of the data indicators [39], combined with regional differences and the actual situation in Xinjiang, this paper selects 24 indicators involving the four aspects of population development level, economic development level, social development level and living environment construction level as the composite index of the urbanization level of Xinjiang counties [40, 41], and the evaluation index system is shown in Table 1.

**Table 1:** Indicator system.

Target	Subsystem	Secondary indicators	Unit
Evaluation Index System of Comprehensive Urbanization in Xinjiang counties	Population development level	The total population	Ten thousand people
		Urban population	Ten thousand people
		Proportion of urban population	%
		Natural population growth	‰
	Economic development level	GDP	Ten thousand Yuan
		Per capita GDP	Yuan
		Total value of industrial products	Ten thousand Yuan
		Local financial income	Ten thousand Yuan
		Expenditure of local finance	Ten thousand Yuan
		The share of primary industry in GDP	%
		The share of secondary industry in GDP	%
		The share of tertiary industry in GDP	%
	Social development level	Fixed asset value of the whole society	Ten thousand Yuan
		Retail volume of social commodities	Ten thousand Yuan
		Average employee’s money wage	Yuan
		Water penetration rate	%
		Gas penetration rate	%
	Living environment construction level	Road area per capita	km^2^
		Drainage pipe density	km/km^2^
		Domestic garbage removal volume	Ton
		Green area rate of built-up area	%
		Coverage rate of afforestation in development area	%
		Per capita public green areas	km^2^
		Public facilities	km^2^

The data are mainly obtained from the *Statistical Yearbook of Xinjiang*, *Xinjiang Urban and County Construction Statistics Annual Report*, reports on the work of the governments of cities and counties, the website of the people’s government of each city, the China Statistical Information Network, and the Xinjiang Uygur Autonomous Region Statistics Bureau of even-numbered years from 1996 to 2018. Some of the missing data are obtained by interpolation of adjacent year values.

#### Entropy method

The calculation of index weights is indispensable in the establishment of a comprehensive evaluation model [42]. There are many methods for calculating the weight of data, such as the analytic hierarchy process, principal component analysis, factor analysis and entropy method. (1) The analytic hierarchy process has more qualitative components and less quantitative data. When there is a large amount of index data, the weights are not accurate enough. (2) Principal component analysis is limited by the cumulative contribution rate standard when processing data, and the extracted principal components have a certain degree of ambiguity. (3) The factor score in the factor analysis method is calculated by the least square method, so there is the possibility of data failure. Relatively speaking, the entropy method can prevent the randomness and uncertainty caused by human factors or dimensionality reduction [43] and the overlap of information contained in the data [44]. Therefore, this article uses the information entropy of the data to determine the weight of the indicator. The main calculation steps are as follows:

First, due to the different dimensions of the indicator data, the selected positive indicators are standardized [45], and the standardized matrix B is obtained. The indicator standardization formula is as follows:

$$X_{ij}^{\prime }=\frac{\left(X_{ij}-\text{min}\{X_{j}\}\right)}{\left(\text{max}\{X_{j}\}-\text{min}\{X_{j}\}\right)},X={\left(X_{ij}^{\prime }\right)}_{n\times m},i=1,2,\cdots ,n,j=1,2,\cdots m$$ (1)

where *X*_*ij*_ is the original value of the *j*-th index of the *i*-th study area, $X_{ij}^{\prime }$ is the standardized value of the index, and min(*X*_*j*_) and max(*X*_*j*_) are the minimum and maximum values of the same index data.

Second, SPSS and Excel software are used to calculate the entropy and weight of the standardized index data value:

1. Calculate the proportion of the *i*-th item under the *j*-th index in the index
$$p_{ij}=\frac{X_{ij}^{\prime }}{\sum_{i=1}^{n}X_{ij}^{\prime }}$$ (2)
2. Calculate the entropy value *e*_*j*_ of the *j*-th index and the information utility value *d*_*j*_
$$e_{j}=-k\sum_{i=1}^{n}p_{ij}\;\text{ln}(p_{ij})$$
$$d_{j}=1-e_{j}$$ (3)
3. Calculate the weights of various indicators
$$w_{j}=\frac{d_{j}}{\sum_{j=1}^{m}d_{j}}$$ (4)

Finally, suppose *a*, *b*, *c* and *d* be the number of indicators in the population, economy, society and living environment system respectively, and *a*_*itk*_, *b*_*jtk*_, *c*_*ztk*_, *d*_*ltk*_ and $X_{ij}^{\prime }$, $X_{jt}^{\prime }$, $X_{zt}^{\prime }$ and $X_{lt}^{\prime }$ are the index weights of the four systems *i*, *j*, *z* and *l* and their corresponding index coefficients, that is, the evaluation models *U*_*1*_, *U*_*2*_, *U*_*3*_ and *U*_*4*_ of the development level of population, economy, society and living environment in Xinjiang county during the *t* period of region *i* are:

$$U_{1}=\sum_{k=1}^{a}a_{itk}\times X_{it}^{\prime },U_{2}=\sum_{k=1}^{b}b_{jtk}\times X_{jt}^{\prime },U_{3}=\sum_{k=1}^{c}c_{ztk}\times X_{zt}^{\prime },U_{4}=\sum_{k=1}^{c}d_{ltk}\times X_{lt}^{\prime }$$ (5)

#### Coupling and coordination model

The coupling model is an evaluation model that measures the degree of influence among multiple systems and whether each system operates in an orderly manner to determine the mutual function and achieve the best benefit [46, 47]. The coupling and coordination degree (CCD) evaluation model can compare multiple systems horizontally with respect to the relative coupling degree and can reflect the development trend of multiple factors changing together in the process of urbanization. The specific implementation steps are as follows:

1. Measurement and calculation of the coupling degree and coordination degree: expand the multisystem capacity coupling coefficient model [48, 49], and establish a population-economy-society-living environment (PESL) coupling model for Xinjiang counties:
$$C={\left\{U_{1}\times U_{2}\times U_{3}\times U_{4}/{\left[\frac{U_{1}+U_{2}+U_{3}+U_{4}}{4}\right]}^{4}\right\}}^{k}$$ (6)
  where *k* is the adjustment coefficient. Because this study needs to conduct a coupling measurement of the PESL, *k* = 4.

Second, establish a model of the coupling and coordination degree of PESL development in Xinjiang counties:

$$D=\sqrt{C\times U},D\in \left[0,1\right],T=\alpha U_{1}+\beta U_{2}+\gamma U_{3}+\delta U_{4}$$ (7)

where *D* is the degree of coordination, *C* is the degree of coupling, *T* is the comprehensive coordination index, and *α*, *β*, *γ*, and *δ* are undetermined coefficients. Because population, economy, society, and living environment have an equally important impact on the development of urbanization, the coefficients are undetermined, so all aspects take the value 1/4. Referring to existing research [50, 51], the coupling coordination state is divided into five levels (Table 2).

**Table 2:** Classification standard and types of CCD.

Coordination State	D value	Corresponding Color
Serious imbalance	D≤0.2	
Imbalance	0.2<D≤0.4	
Basic coordination	0.4<D≤0.6	
Coordination	0.6<D≤0.8	
Good coordination	0.8<D≤1	

#### Local spatial autocorrelation analysis

Spatial autocorrelation statistics are used to measure a basic property of geographic data: the degree of interdependence between a variable at a certain position in a space and the data of adjacent variables [52]. This dependence is usually called spatial dependence. Due to the influence of spatial interaction and spatial diffusion, geographic data may no longer be independent of each other but become related [53]. Spatial autocorrelation statistics are widely used in geostatistics. There are a variety of indexes that can be used, but the most important ones are Moran’s *I* and Geary’s C. The functions of these methods are roughly divided into two categories: global and local hypothesis testing [54]. The formulas are as follows:

1. Global spatial autocorrelation analysis
$$I=n\sum_{i=1}^{n}\sum_{i=1}^{n}w_{ij}(x_{i}-\bar{x})/{\left[\sum_{i=1}^{n}\sum_{i=1}^{n}w_{ij}(x_{i}-\bar{x})\right]}^{2}$$ (8)
2. Local spatial autocorrelation analysis
$$I=\frac{\left(x_{i}-\bar{x}\right)}{S^{2}}\sum_{i\neq j}^{}w_{ij}(x_{i}-\bar{x})$$ (9)
  where *I* is the local autocorrelation index of county *i*, and *I* refers to the coupling coordination index in this study.

Global spatial autocorrelation analysis can fail to describe the overall distribution of the phenomenon and judge whether the phenomenon is clustered in space, but it cannot exactly show the clustering in those areas. Based on the local indicators of spatial association (LISA) methodology proposed by Anselin [55] in 1995, this paper analyzes the spatial aggregation phenomenon and spatial correlation of the coupling and coordination of 76 counties in Xinjiang. Using the local spatial autocorrelation analysis in the ArcGIS spatial data analysis module, four types of aggregation can be constructed [56, 57]: High-High cluster (H-H), Low-High cluster (L-H), Low-Low cluster (L-L) and High-Low cluster (H-L), which represent the counties in 76 county diffusion and mutual spillover zones, polarization benefit zones, low-speed growth zones and backward transition zones, respectively [58].

**Fig 2:**
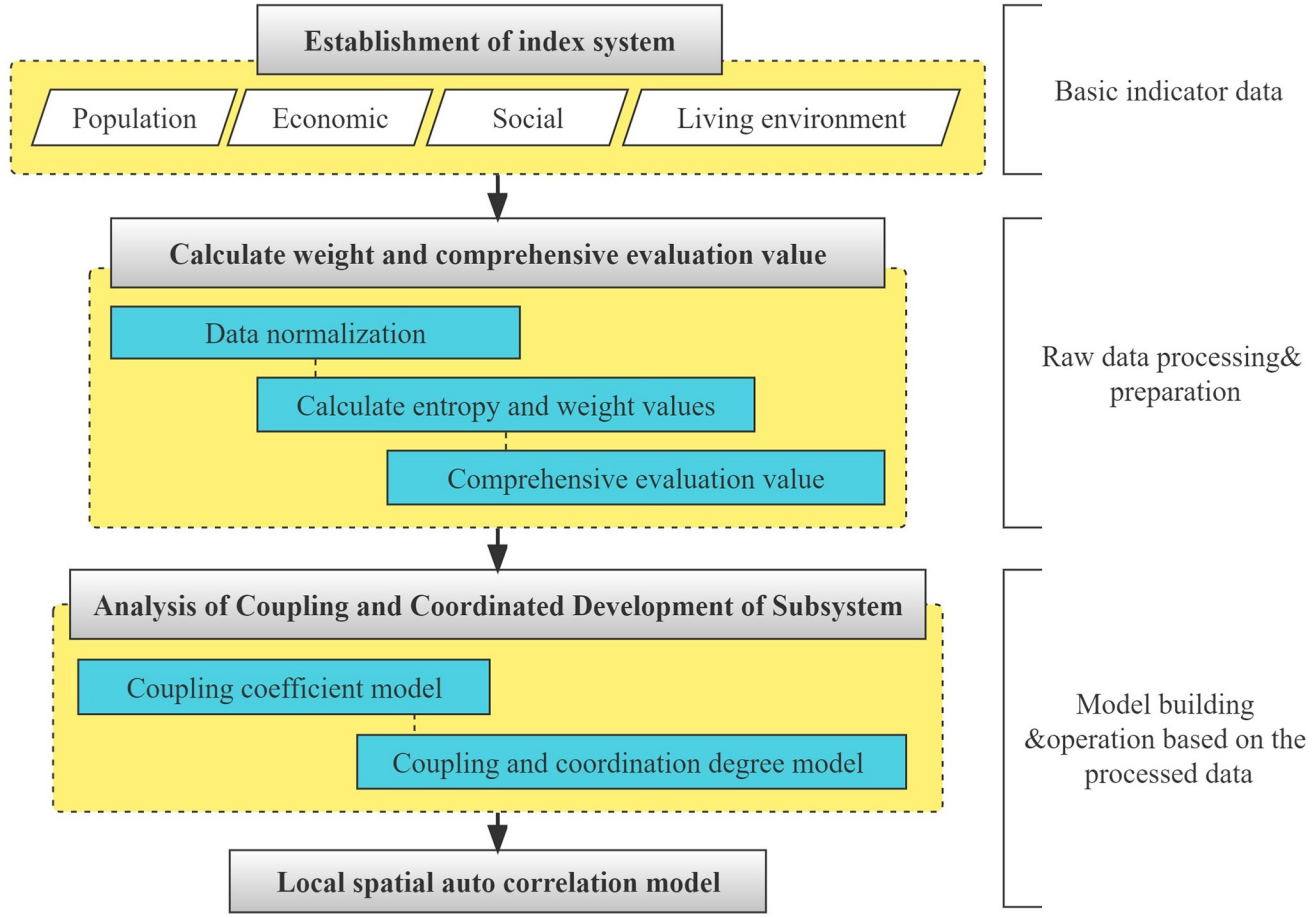
Methodological flowchart.

The application framework of the main methods in the paper is shown in Fig 2:

## Results

### Analysis of the comprehensive urbanization development level

**Fig 3:**
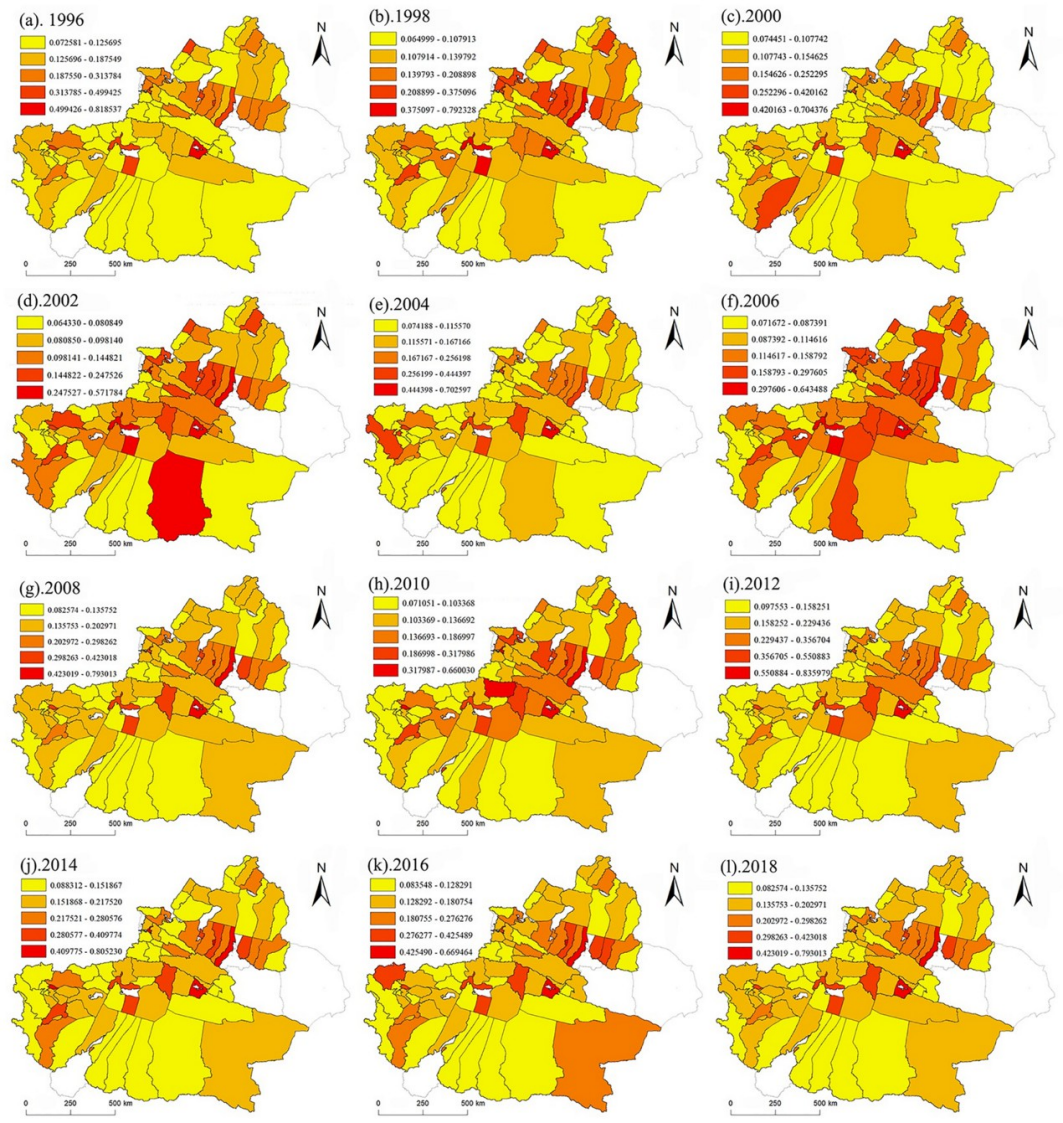
The distribution of comprehensive urbanization levels in XJC from 1996 to 2018. ^a^ The blank areas in the figure are cities and counties that are not included in this study.

**Fig 4:**
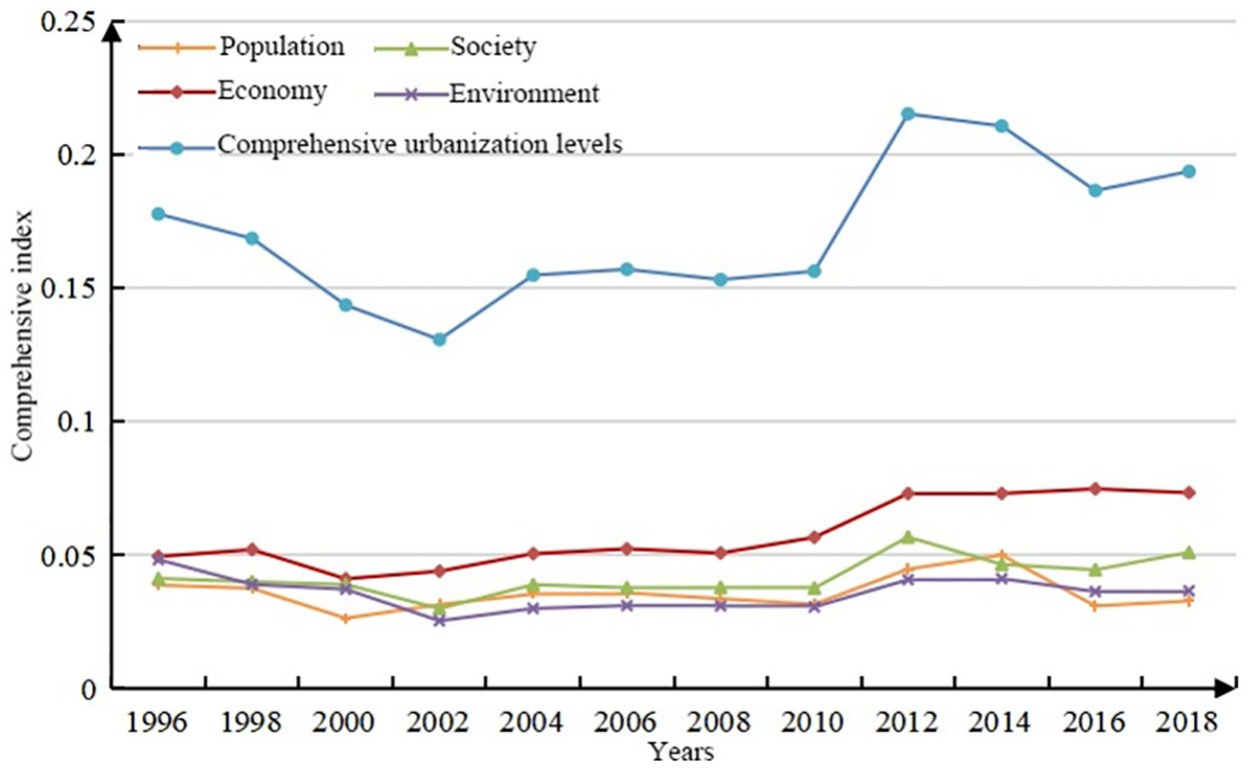
The development trend of the comprehensive evaluation index of XJC urbanization from 1996 to 2018.

From the above comprehensive urbanization evaluation model, the comprehensive urbanization evaluation index of 76 counties in Xinjiang from 1996 to 2018 was obtained, and ArcGIS was used to visualize the temporal and spatial distribution of urbanization development. The results are as follows:

It can be seen from Fig 3 that since 1996–2018, the comprehensive urbanization level of 76 counties in Xinjiang has shown obvious "center-periphery" development characteristics. The counties with a high level of comprehensive urbanization are mainly located on the northern slope of Tianshan Mountain, and the level of comprehensive urbanization decreases with distance. (1) Shihezi, Changji, Fukang, Usu, Yining, Kuitun and Manas in the northern Xinjiang region are at a higher level (0.231~0.485). Altay, Bole, Jimusaer, Mulei, Hutubi, and Shawan are at the middle level (0.212~0.217), and the remaining counties in northern Xinjiang are at a lower level. (2) Korla, Aksu, Hotan, and Kuqa in southern Xinjiang are at a higher level (0.212~0.723). and Artux, Hejing, Shache, etc., are in the middle (0.230~0.175). The rest of the southern Xinjiang counties are at a lower level. In general, the comprehensive urbanization level of Xinjiang’s counties showed a slow rise from 1996 to 2018, a rapid rise from 2010 to 2012, and a slow decline thereafter. In 2012, the county’s comprehensive urbanization level showed the highest level, and the economic, social and living environment subsystems reached the highest value, while the population development reached the highest value in 2014 (Fig 4). The social and living environment subsystems have a certain similarity with the comprehensive urbanization level curve, indicating that the development of society and living environment has a large impact on the urbanization of Xinjiang counties. This shows that in the process of urbanization development, the gathering of the population in cities or urban population growth cannot be defined as the driving force of urbanization development, but it also comes from the coordinated development of social and living environment factors.

### The distribution of the PESL coupling coordination

**Fig 5:**
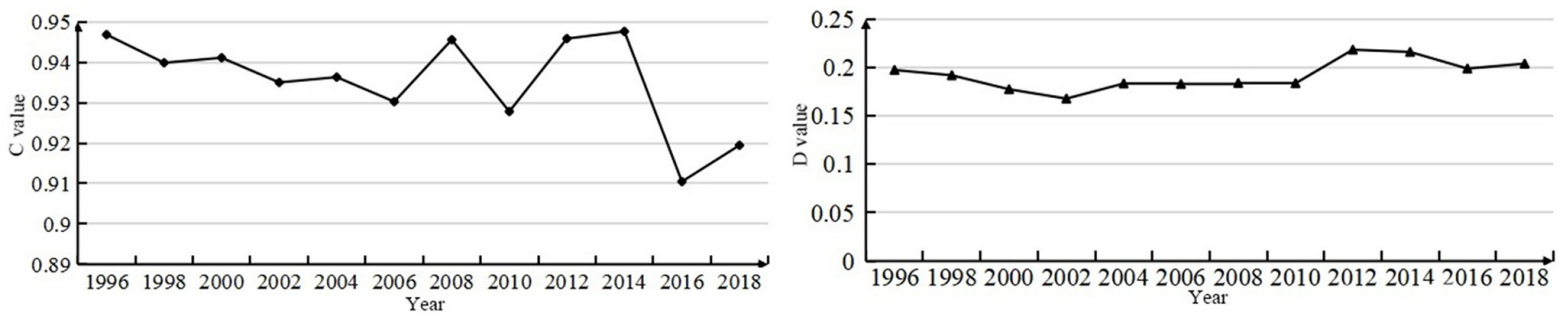
The development trend of the coupling and coordination of PECL in XJC from 1996 to 2018. ^a^ The picture on the left shows the development trend of the coupling situation, and the picture on the right shows the development trend of the coupling coordination situation.

**Fig 6:**
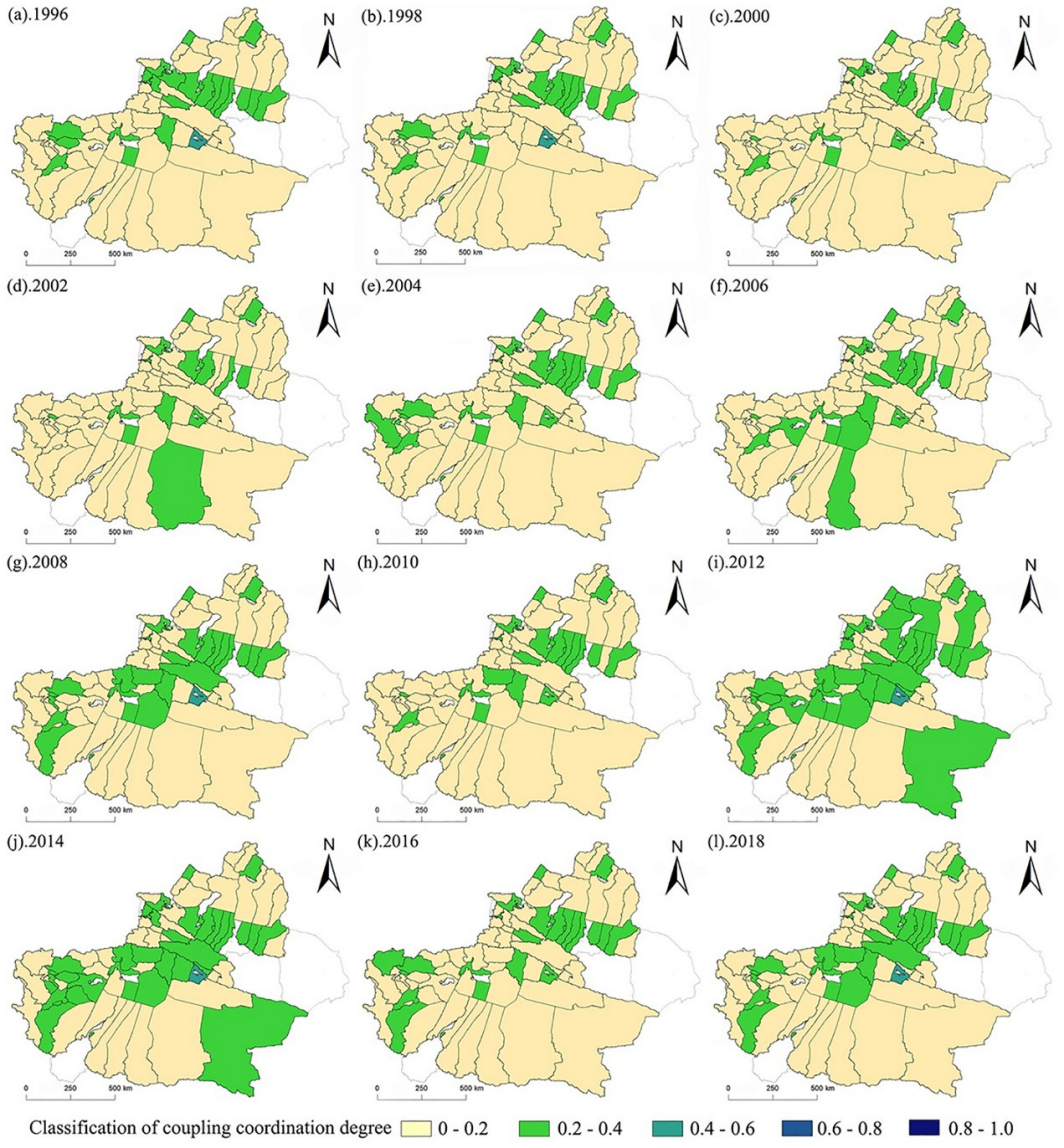
Coupling and coordinated temporal and spatial distribution of PECL in XJC from 1996 to 2018.

Based on the above comprehensive urbanization evaluation index, the average PESL coordination degree of 76 counties in Xinjiang from 1996 to 2018 is obtained (Fig 5) to analyze the development trend:

From the analysis of the degree of coupling and coordination of 76 counties in Xinjiang, there are large differences in coupling and coordination. During the period from 1996 to 2018, the integrated urbanization coupling situation of Xinjiang counties showed an unstable development trend. The coupling coordination can be analyzed in three stages, as follows:

From 1996 to 2002, the coupling coordination of integrated urbanization showed a downward trend. This may indicate that the various factors are not sufficiently coordinated, which hinders the overall development of urbanization and makes the urbanization process of Xinjiang Counties lag behind. (2) From 2002 to 2012, comprehensive urbanization showed a slow upward trend, indicating that the coordination between the various subsystems gradually developed during 2004–2010, it rose in an all-round way in 2012, and the comprehensive urbanization level showed a good development trend. (3) During the period from 2010 to 2018, comprehensive urbanization development showed an upward trend in 2018 after presenting a lagging phenomenon again. To observe the coupling and coordination distribution of 76 counties in Xinjiang more intuitively, Fig 6 shows the following:

It can be seen from Fig 6 that only Korla is in the third class (0.4–0.6) in all years, and the coupling coordination degree shows a low degree of coordination. Usu, Shawan, Shihezi, Changji, Fukang, Altay, Tacheng and Yining in the northern Xinjiang region and Manas, Aksu, Atushi, Hotan, Xinyuan, Kuqa, Shache, Yecheng etc., in the southern Xinjiang region are in the fourth class (0.2–0.4), and the degree of coupling coordination presents an imbalance. Most of the other cities and counties in Xinjiang are at the lowest level (0–0.2). In conclusion, the degree of urbanization coupling coordination among counties in Xinjiang, except for the counties and cities near the northern slope of the Tian Mountains, presents a serious imbalance, especially the degree of PESL coupling and coordination in counties and towns in the border zone, which is not in an ideal state.

### Local spatial distribution of the degree of coupling and coordination

Using the PESL coupling coordination index, based on ArcGIS local spatial autocorrelation analysis, the local spatial pattern of the coordinated development of 76 counties in Xinjiang from 1996 to 2018 is analyzed.

**Fig 7:**
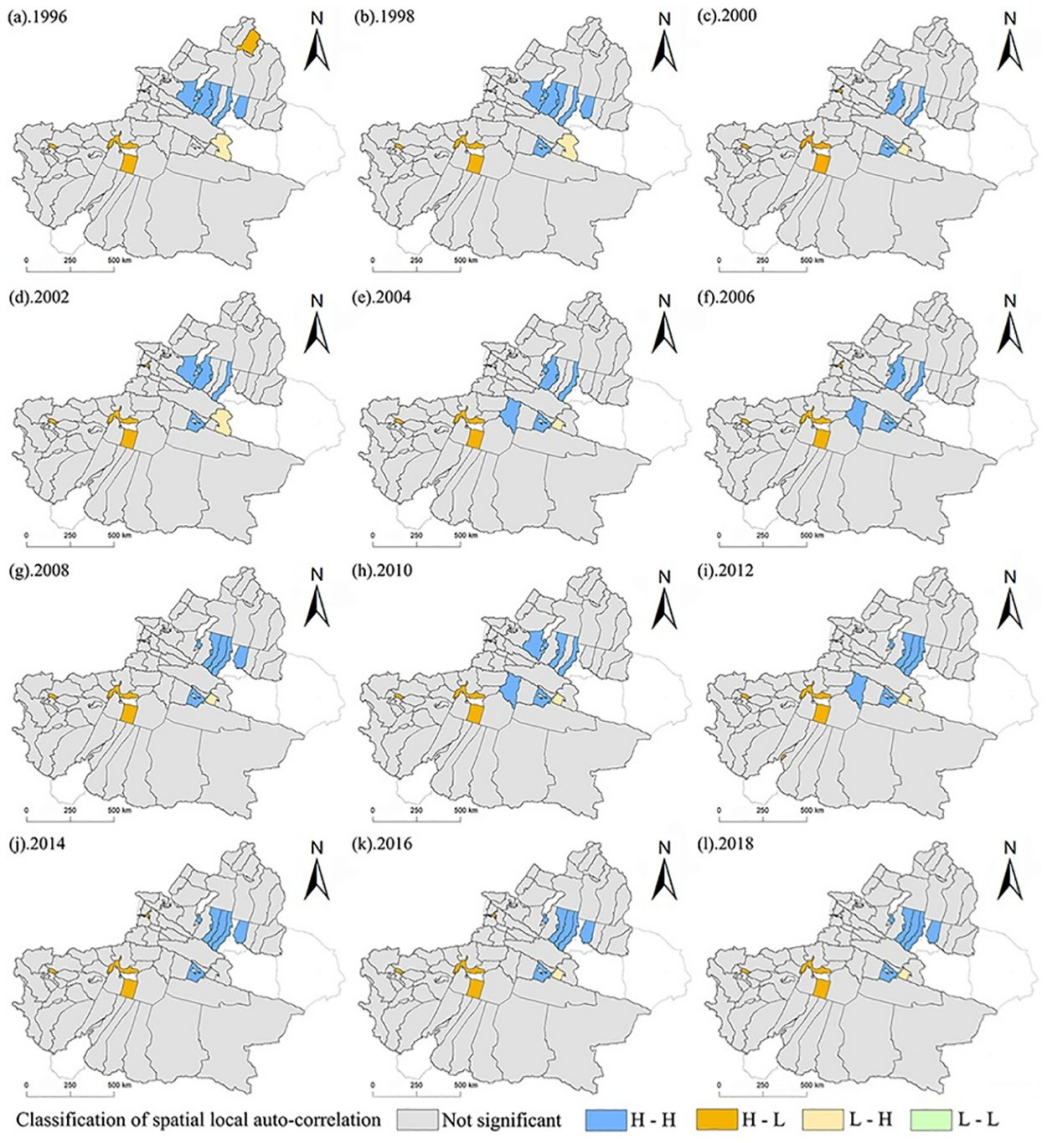
LISA map of coupling coordination in Xinjiang counties from 1996 to 2018.

Most of the spatial distribution of the PESLcoupling coordination index of 76 counties in Xinjiang in the process of urbanization showed no spatial correlation, a few counties showed high-high agglomeration, and very few showed a high-low agglomeration state. From 1996 to 2018, Shihezi, Changji, Fukang, Hutubi, Manas, Kuitun around Urumqi and Korla in the southern Xinjiang region showed high-high agglomeration status. These account for 12.5% of all counties, which means that these counties are adjacent to the surrounding counties with high attributes and form a diffusion and mutual overflow area. For example, Aksu, Kashgar, and Hotan in the southern Xinjiang region show high-low aggregation, accounting for 3.94%. This shows that the low attribute values of these small counties are adjacent to the high attribute values of surrounding counties, which are polarized benefit areas. The remaining 83.56% of counties show no spatial correlation, indicating that most counties in Xinjiang have no attribute value adjacent to the surrounding counties, and there is no mutual overflow effect. Combined with Fig 7 above, most counties in Xinjiang are still in a state of imbalanced PESL coupling and coordination, and their urbanization development is not strong enough.

## Discussion

1. According to the analysis results of urbanization level, the overall comprehensive urbanization level index of northern Xinjiang is higher than that of southern Xinjiang. This is consistent with the fact that the social and economic development level of northern Xinjiang has been higher than that of southern Xinjiang for a long time [59]. This is also closely related to the development of Xinjiang’s high priority cities (Urumqi, Karamay, etc.) have developed and have weak external links [60], the polarization between the leading city and small cities and counties is strong, and the radiation driving effect is not obvious. Fig 3 shows that the comprehensive urbanization level of the economic belt on the northern slope of the Tian Mountains is relatively high, and the further it extends, the lower the comprehensive urbanization development level. First, the climate north of Tian Mountain in Xinjiang is suitable, the primary industry is concentrated there, and the main prefecture-level cities in Xinjiang are basically located there. Second, Xinjiang’s main traffic arteries, highways, and railway stations are mostly located near the economic belt on the northern slope of the Tian Mountains, so this area has become an important intersection along the New Silk Road and a key route for the economic, cultural, and logistics interaction between Xinjiang and inland China. However, among the 76 counties in Xinjiang, Korla has the highest comprehensive urbanization index (0.7225), indicating that it is also at the forefront in economic development, social development and living environment construction. Due to the long distance between other counties in southern Xinjiang and those in the economic belt on the northern slope of the Tian Mountains, the inconvenient transportation and the ecological environment is sensitive and fragile, the comprehensive urbanization level is still unsatisfactory, and the development level is relatively backward, resulting in the weakening of the radiation driving effect of a few county-level cities on the surrounding counties [61]. Especially for the border counties, urbanization development has been seriously hindered.
2. Fig 5 shows that the coupling and coordination of the PESL in Xinjiang counties is steadily developing. However, it can be seen more directly from Fig 6 that only Korla (the average is 0.3948) consistently has a high value. Korla is the capital city of Bayingoleng Mongolian Autonomous Prefecture, GDP is often in the first place of per capita GDP in Xinjiang,. Korla’s rich petroleum resources and rationalized tourism resources drive the industrial development of surrounding counties [62], and the development speed of Korla is significantly higher than that of other areas in southern Xinjiang. However, southern Xinjiang has a vast territory, accounting for 65% of the total area of Xinjiang, and a central city alone cannot drive the overall urbanization development of the region. For example, the integrated urbanization coupling coordination status of Aksu, Atushi, Hotan (with averages of 0.3004, 0.2042 and 0.2298) and other counties is in a moderately unbalanced state, and the counties surrounding the three are in a severely dysregulated state. The counties in southern Xinjiang are small and far away, and given the geographical location south of the Tian Mountains, the natural environment in southern Xinjiang is dominated by drought and salinity. Due to the uneven distribution of economic development, capital, technology and labor force in the southern and northern counties, the coupling and coordination of the PESL in the southern counties, especially along the border counties, is irrelevant.

## Conclusion

1. The comprehensive urbanization level of 76 counties in Xinjiang presents obvious "center-periphery" development characteristics. The counties with higher comprehensive urbanization levels are mainly located on the northern slopes of the Tian Mountains. The level of comprehensive urbanization decreases with distance from this area.
2. From 1996 to 2018, the population, economy, society and living environment of the counties and cities near the northern slope of the Tian Mountains developed steadily. In addition, the other counties were in a serious imbalance situation, especially in the population, economy, society and living environment of the counties and towns in the border area was not ideal and stable for many years.
3. Among the 76 counties in Xinjiang, Shihezi, Changji, Fukang in the northern Xinjiang region and Korla in the southern Xinjiang region belong to the diffusion and overflow areas. These counties drive the surrounding cities to form a concentrated development area; Aksu, Kashgar and Hotan belong to the polarization benefit area. These counties can improve the comprehensive urbanization development of the region by absorbing the population and economy of the surrounding counties; Most of the other counties are in the state of no spillover effect, which indicates that more than 63 counties in Xinjiang are still in the state of unbalanced development of population, economy, society and living environment, and their comprehensive urbanization development also presents a low trend.

In the process of county urbanization in Xinjiang, the government should adopt the form of "point-axis development", give priority to the development of counties around regional capital cities and counties along railways, and influence the development of surrounding counties through the radiation of the central county. Xinjiang has a vast territory, with a large number of counties and scattered distribution, and the development of urbanization has obvious regional differences. In the process of urbanization, the actual situation of each county should be accurately grasped. People flow and logistics hubs should be built between distant counties. Through promotion and publicity, the popularization rate of local modern scientific and technological knowledge should be increased. Modern agricultural and industrial advanced technologies should be introduced in central China to improve and upgrade traditional agriculture and industry, thereby fostering the leap-forward development of characteristic industries in the counties. For coastal counties, while ensuring national security, localities should make full use of the state’s policy of opening to the outside world to promote cultural and logistics exchanges between Xinjiang’s border counties and neighboring countries and push the development of tertiary industry in coastal counties to coordinate the development of regional urbanization.

There are still some deficiencies in the research of this paper. The indicators used in the study are selected based on the actual situation of Xinjiang counties and the principles of the completeness, universality and availability of data. Therefore, there is a lack of indicator data reflecting the income and expenditure of residents, medical care, education and infrastructure penetration, etc., it fails to fully consider the constraints that have an impact on the urbanization of Northwest China, such as county water resources, railways, transportation and oasis economy, which has a certain impact on the research results. In subsequent research, in the context of the new urbanization construction policy and under the principle of data reliability, data indicators related to the residential environment, residents’ disposable income, and infrastructure construction will be updated and added. In terms of research methods, the results of the coordinated coupling of comprehensive urbanization will be further verified and predicted to enrich the research content regarding the coordinated development of comprehensive urbanization in Xinjiang.

## Supporting information

S1 Data (ZIP)
